# Mechanism of the enhanced coercivity for the dual-main-phase Ce–Fe–B magnet

**DOI:** 10.1038/s41598-020-75082-w

**Published:** 2020-10-21

**Authors:** Rui Han, Hongsheng Chen, Dong Zhou, Xiaoning Shi, Jiyuan Xu, Shengzhi Dong, Minggang Zhu, Wei Li

**Affiliations:** Division of Functional Material, Central Iron and Steel Research Institute, Beijing, 100081 People’s Republic of China

**Keywords:** Materials science, Condensed-matter physics, Magnetic properties and materials

## Abstract

The high coercivity of Nd–Fe–B magnets can also be obtained in the Ce–Fe–B magnets fabricated via the dual-main-phase (DMP) method in which the high abundance Ce was used to substitute Nd(Pr). The inhomogeneous distributions of the matrix grains in the DMP magnet play a key role in the enhanced magnetic performance. Compared with the single-phase magnet, more grain boundary phases encapsulating the matrix 2:14:1 grain are formed in the DMP magnet, which reduce the exchange coupling between adjacent magnetic grains. The switching field distribution and the interaction field distribution of the Ce–Fe–B magnets were determined by the first-order-reversal curves (FORC). The switching field peaks around 6 kOe, 11 kOe and 12 kOe in the FORC distribution indicate that three major reversal components coexist for the DMP magnet. The overlapp of the second and third switching field peaks reveals the presence of a pinning interaction within individual magnetic grains with a core–shell structure, which further improve the coercivity of the magnet.

## Introduction

Permanent magnets are critical components for numerous devices ranging from traction motors for hybrid electrical vehicles to wind generators. In particular, magnets with 2:14:1 type Nd–Fe–B alloys exhibit excellent properties and are now widely used in many fields^1^. The rapidly increasing demand for Nd–Fe–B magnets along with the Pr/Nd supply restrictions, make a strong economic case for developing competitive magnets. From commercial standpoints, less expensive and more abundant isostructural compounds with similar magnetic properties are always desired, so various isostructural Re_2_Fe_14_B compounds have been investigated and their properties analyzed^2–4^. In particular, the large backlog and inexpensive Ce-based magnets are currently research hotspots^5–11^.

It is well known that the saturation magnetization and anisotropy field of Ce_2_Fe_14_B (1.2 T and 3600 kA/m, respectively) are about 25% and 36% lower than those of Nd_2_Fe_14_B (1.6 T and 5600 kA/m, respectively). Okada et al*.* have prepared sintered (Nd_0.8_Pr_0.15_Ce_0.05_)–Fe–B magnet with a maximum energy product (*BH*)_*max*_ and coercivity *H*_*cj*_ of 39.9 MGOe and 10.2 kOe, respectively^4^. Zhou et al*.* reported achievement of a sintered (Nd_0.5_Pr_0.1_Ce_0.4_)–Fe–B magnet with *H*_*cj*_ = 9.2 kOe and (*BH*)_*max*_ = 28.3 MGOe^12^. The lower magnetic properties of Ce-based magnets prepared by the traditional sintering method limit the Ce–Fe–B as a possible alternative to Nd–Fe–B. To solve this problem, our group proposes the use of a dual-main-phase (DMP) method, which can push the Ce content to a high level with moderate magnetic properties^8,13–15^. The DMP magnet possesses higher values of coercivity and rectangularity compared to the single-phase (SP) magnet prepared by the traditional sintering method^13^.

The coercivity for sintered Nd–Fe–B magnets is highly sensitive to the intrinsic magnetic properties of 2:14:1 phase and the microstructure of the magnet^5,16^. The low magnetocrystalline anisotropy close to defects and the short-range exchange couplings between the non-isolated matrix grains lead to lower coercivity than the theoretical *H*_*A*_, and this is known as the Brown’s paradox^17,18^. Note that the above principle relies on the assumption that the Nd_2_Fe_14_B matrix phase is homogeneous either within an individual grain or among all the grains. However, for DMP magnet, which is developed by mixing Ce-free and Ce-containing grains, the matrix phases are inhomogeneous within an individual grain and among all the grains. The interior interactions of local regions within an individual main phase grain and the exchange coupling among different grains in DMP magnets may coexist, which will influence the coercivity of the magnet. This work focuses on the reversal mechanism for the DMP magnet, where the microstructural features and the interior interactions were used to explain the enhanced coercivity of the DMP magnet.

## Results and discussion

**Figure 1:**
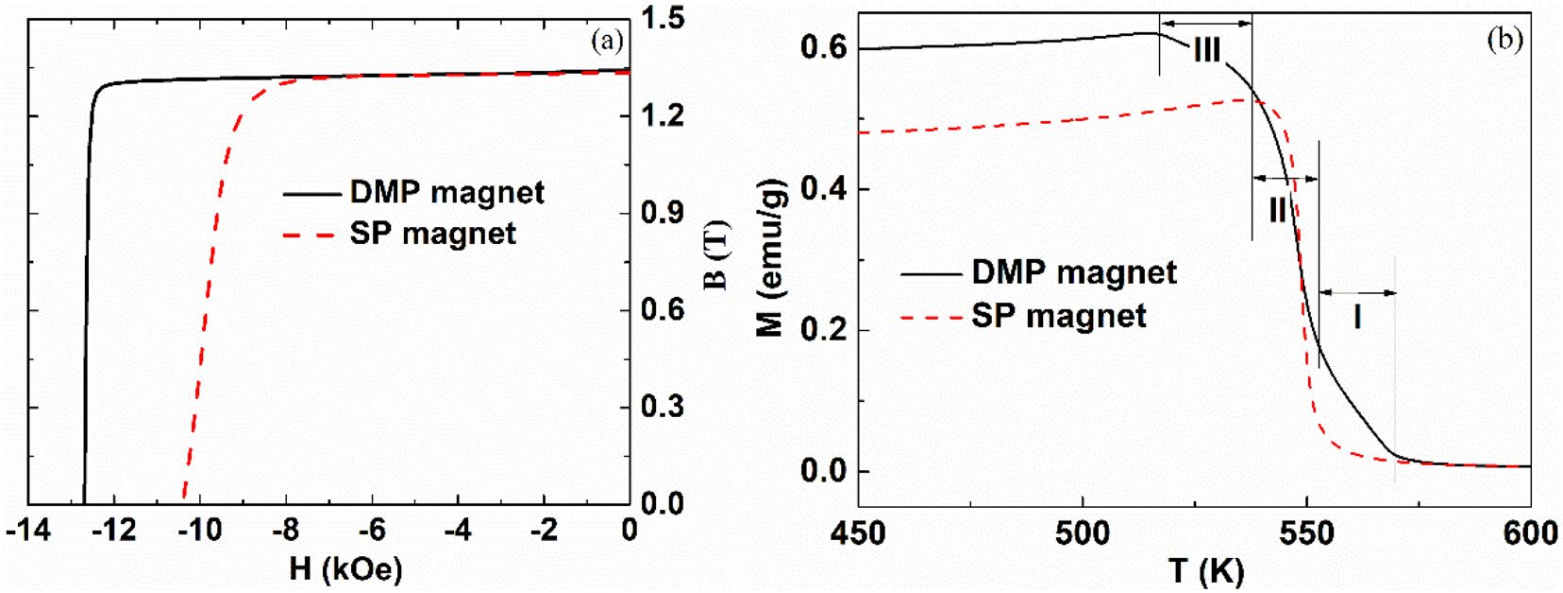
Demagnetization curves (**a**) and temperature dependence of magnetization curves with a magnetic field of 0.05 T (**b**) for the DMP and SP magnets.

The demagnetization curves for the as-sintered DMP and SP magnets are shown in Fig. 1a and the magnetic parameters obtained from Fig. 1a, are shown in Table 1. As it can be seen the coercivity and the rectangularity of the DMP magnet are much larger than those of the SP magnet prepared by the traditional method, while the remanence values are almost the same. The coercivity and the rectangularity of the DMP magnet reach 12.7 kOe and 0.95, respectively.

**Table1:** Magnetic properties of the as-sintered samples employed in the present study.

Magnet	Remanence (kGs)	Coercivity (kOe)	(BH)_max_ (MGOe)	Rectangularity
DMP magnet	13.4	12.7	41.9	0.95
SP magnet	13.3	10.4	40.2	0.86

Figure 1b shows the temperature dependence of the magnetization curves with a magnetic field of 0.05 T for both samples. In contrast to SP magnet with one single Curie temperature phase, it is evident that DMP magnet possesses three Curie temperature phases. Moreover, the second Curie temperature is almost the same with that of the SP magnet. It is well known that increasing the cerium content results in reduction of the Curie temperature^19^, and hence these three kinds of matrix phases may correspond to the (Pr/Nd)_31_(Fe,TM)_68_B_1_, (Pr/Nd_0.8_Ce_0.2_)_31_(Fe,TM)_68_ B_1_ and (Pr/Nd_0.5_Ce_0.5_)_31_(Fe,TM)_68_B_1_ grains.

**Figure 2:**
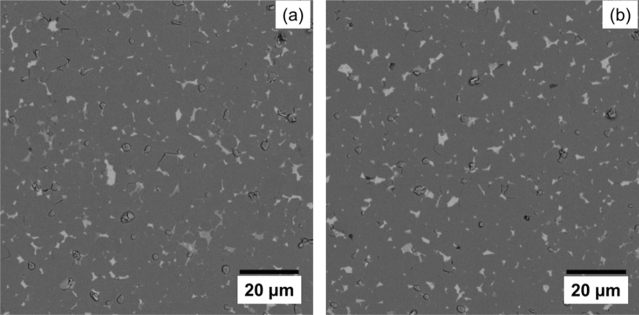
BSE-SEM images of the DMP magnet (**a**) and SP magnet (**b**).

**Figure 3:**
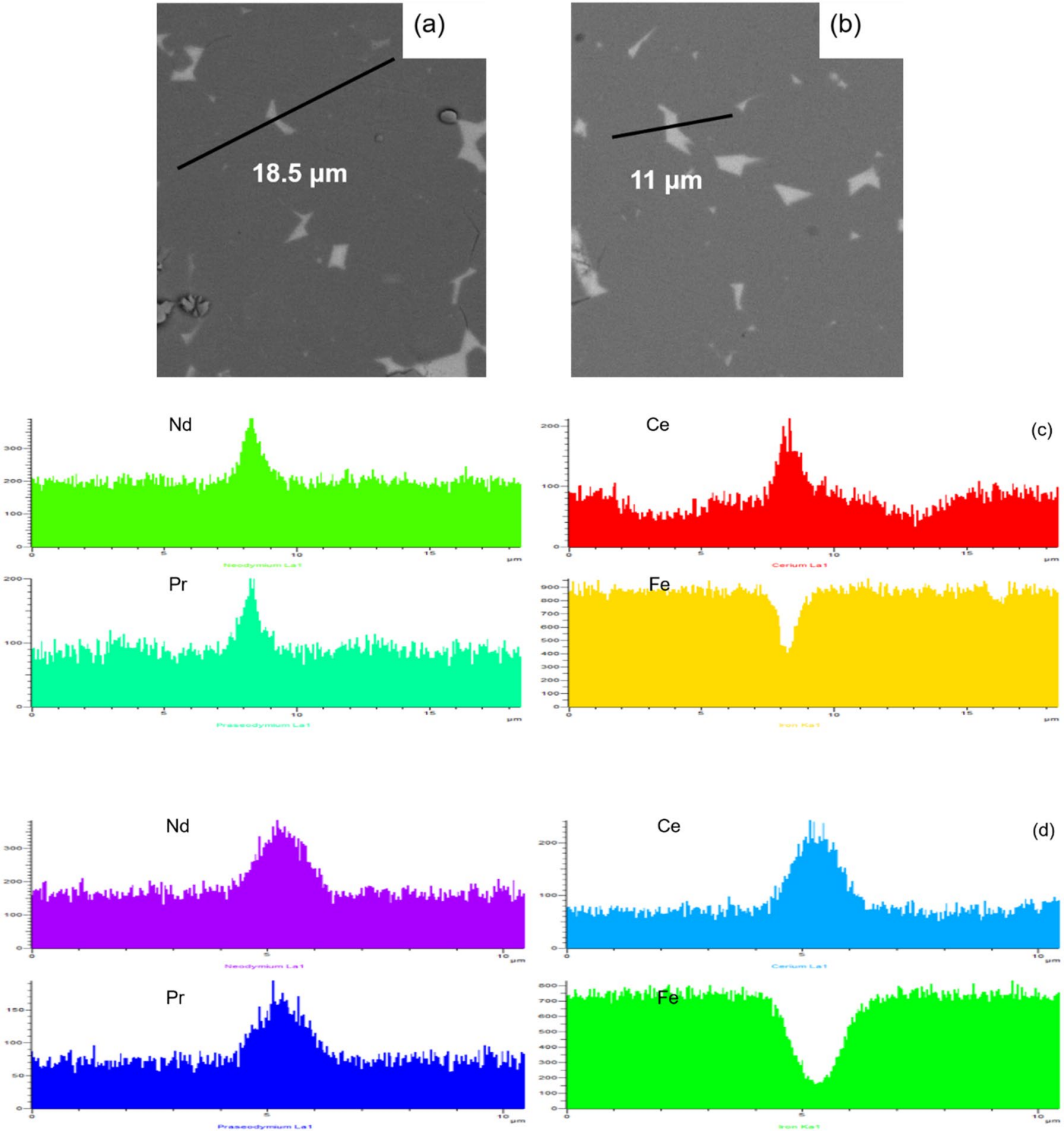
SEM images of the as-sintered DMP magnet (**a**) and SP magnet (**b**). (**c**) EDS spectra in (**a**) and (**d**) EDS spectra in (**b**).

Figure 2 depicts backscattered SEM images of the as-sintered magnets. The dark gray regions are the matrix phases, and the brightly imaged regions refer to the so-called “grain boundary phase”. This figure illustrates that the grain boundary phase tends to encapsulate the matrix phase for the DMP magnet, which will reduce the exchange coupling between adjacent matrix grains and improve the coercivity, while the grain boundary phase is prefer to aggregate at the triple junction areas for the SP magnet. In order to identify the distribution of the matrix phases around the grain boundary phase, the Nd, Ce, Pr and Fe elemental profiles perpendicular to the grain triple junction for both magnets, were obtained via EDS and shown in Fig. 3. For both magnets, the triple junction areas show an enrichment of RE (Nd, Ce and Pr) elements, while Fe is lower in these areas. It is worth noting that, for the DMP magnet, the Ce profile is distributed inhomogeneously across the matrix grains, which is different from the distribution of Ce elements for the SP magnet. This gradient distribution indicates that the matrix phases contain various Ce-containing grains. Moreover, the magnitude profile of Ce in the matrix phase shows that there is penetration of Ce from the grain boundary into the (Pr/Nd)–Fe–B (alloy I) grains during the sintering process and an obvious core–shell structure is obtained for the DMP magnet^5,6,20^.

**Figure 4:**
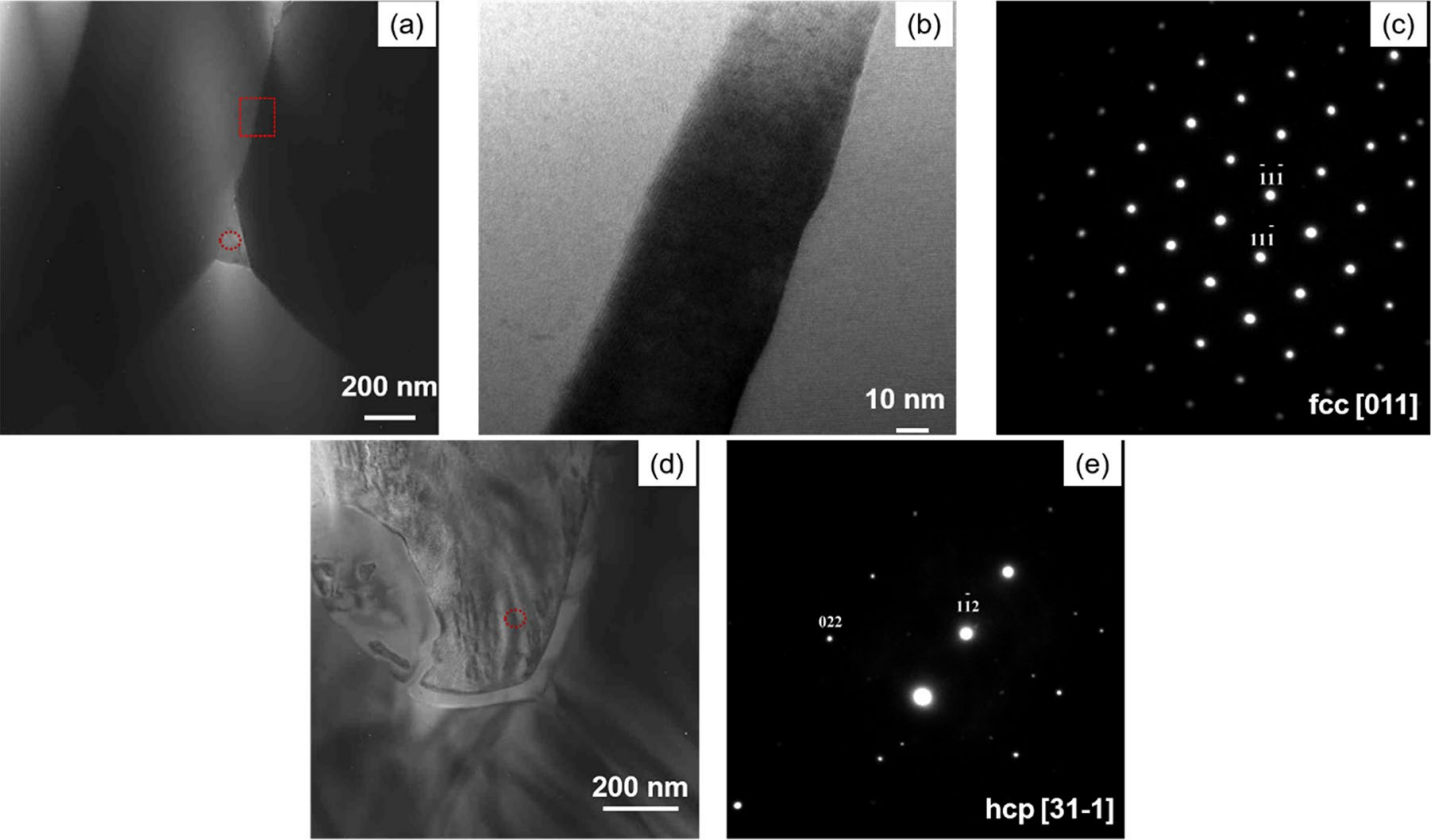
(**a**) Bright-field TEM image of the triple junction in the DMP magnet, (**b**) magnified image of the rectangular region indicated in (**a**), (**c**) SAED pattern along [011] zone axis of the oval region at the triple junction in (**a**), (**d**) bright-field TEM image of the triple junctions in the SP magnet, and (**e**) SAED pattern along [31-1] zone axis of the oval region at triple junction in (**d**).

To characterize the crystalline structure of the grain boundary phase, bright-field TEM images of these areas in the DMP magnet are obtained and shown in Fig. 4a,b. As shown in Fig. 4b, the thickness of the grain boundary phase is approximately 40 nm. The selected-area electron diffraction (SAED) pattern along [011] zone axis of the triple junction phase in Fig. 4c reveals that the triple junction connecting to the grain boundary has a face center cubic (fcc) structure, and the lattice parameter is 0.55 nm, which is corresponds to fcc-(Ce, Nd, Pr)O_x_. The formation of this metastable (Ce, Nd, Pr)O_x_ is thought to be due to the oxidation of the metallic (Ce, Nd, Pr), which minimizes the excess interfacial energy with the matrix grains^21–23^. The RE-rich phase in the Ce_2_Fe_14_B magnet has a lower eutectic reaction temperature and better wetting characteristics than those of Nd_2_Fe_14_B^7,15^, hence, the wettability of the triple junction phases improves dramatically with increasing Ce content. During sintering process, the liquid RE-rich phase of the DMP magnet at the triple junction is easily pulled into the grain boundaries by capillary action to form a grain boundary phase encapsulating the matrix 2:14:1 phase, as shown in Fig. 4a. The coercivity is reported to be simultaneously improved with the formation of grain boundaries due to the reduction of the exchange coupling between adjacent matrix grains. Figure 4d, e depict the bright-field TEM images of the triple junctions and the SAED pattern along [31-1] zone axis of the triple junction in the SP magnet. It is obvious that the RE-rich phase is only distributed in the triple junction regions, while the adjacent main phase grains appear to be connected. From the SAED pattern, the triple junction phase can be indexed to a hexagonal close-packed (hcp) structure with the lattice parameters of a = 0.386 nm and c = 0.604 nm, which is induced by the low oxygen level in the RE-rich phase and has an adverse effect on the coercivity of the magnet^24^.

To investigate the switching field distribution as well as the interaction distribution of the DMP magnet, the first-order-reversal curve (FORC) diagrams were obtained and analyzed. The magnetization was measured from saturation to a reversal field, defined as *H*_*r*_, and then back to the positive saturation field to produce a FORC. A family of FORC curves, obtained with different *H*_*r*_, fill the interior of the major hysteresis loop. According to the classical Preisach model, the FORC distribution $\rho ({\text{H}}_{{\text{a}}} ,{\text{H}}_{{\text{r}}} )$ is defined as follows^25^:

1 $$ \rho ({\text{H}}_{a} ,{\text{H}}_{r} ) = - \frac{1}{2}\frac{{\partial^{2} m(H_{a} ,H_{r} )}}{{\partial H_{a} \partial H_{r} }}, $$

where *H*_*a*_ is the applied field and *m*(*H*_*a*_*, H*_*r*_) is the measured magnetization of the sample at applied field *H*_*a*_ on the FORC with reversal field *H*_*r*_. *ρ* can be seen as a function of the local coercivity *H*_*c*_ and bias field *H*_*u*_ after a coordinate transformation: *H*_*u*_ = (*H*_*a*_ + *H*_*r*_)*/2* and *H*_*c*_ = (*H*_*r*_* − H*_*a*_)*/2*. The *H*_*a*_ and *H*_*r*_ for the FORC measurements for the DMP and SP magnets were both 300 Oe.

**Figure 5:**
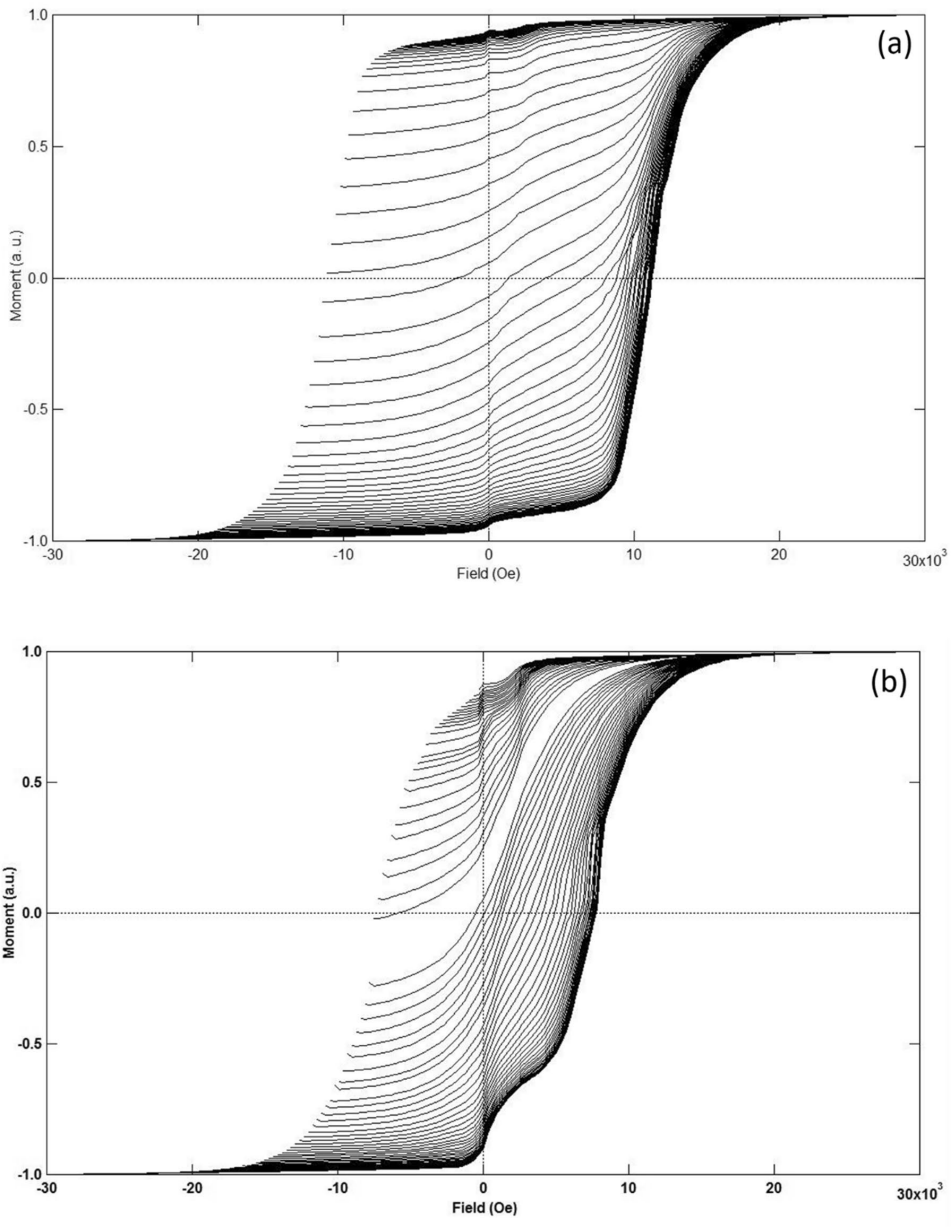
Original FORCs of the DMP magnet (**a**) and SP magnet (**b**).

**Figure 6:**
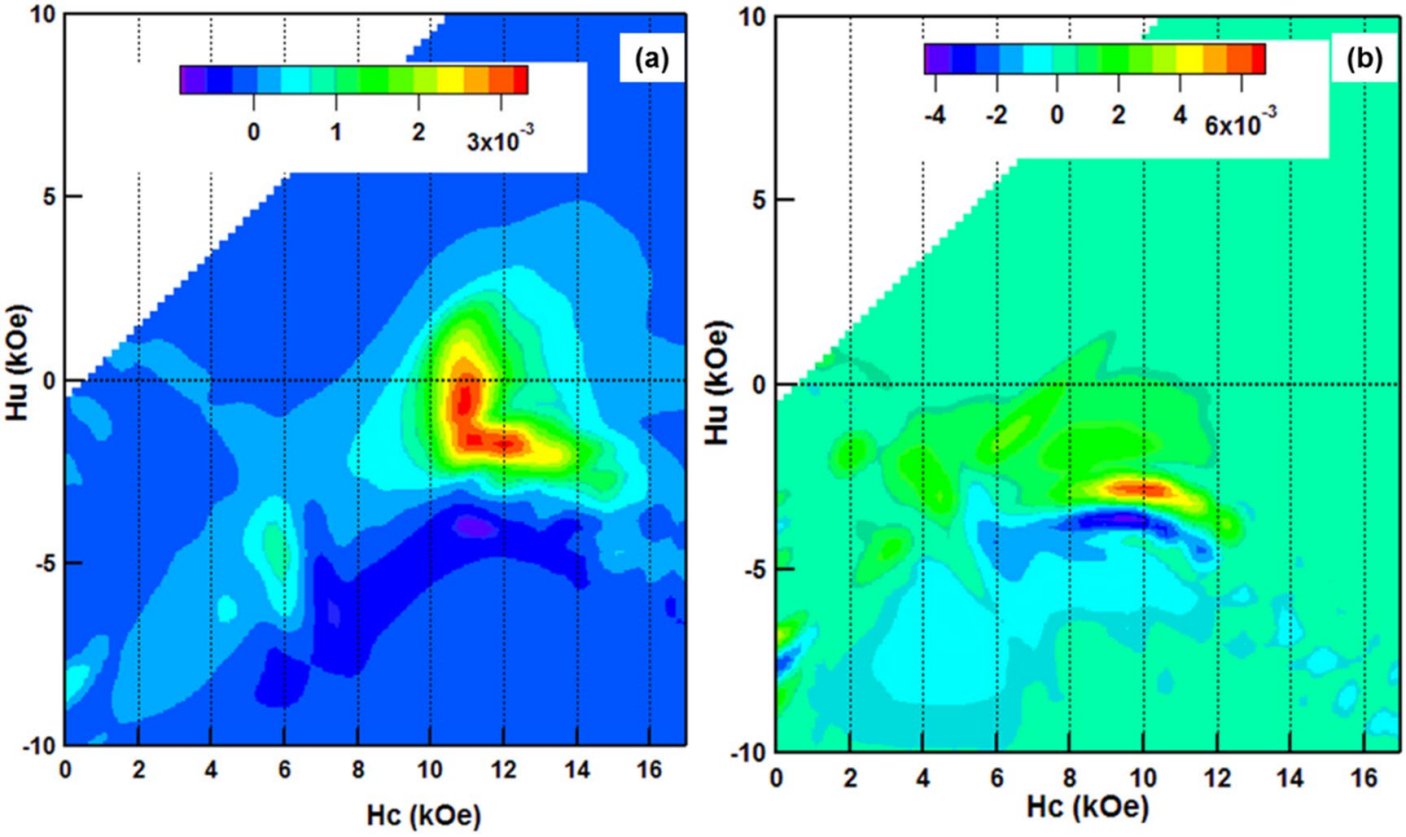
Converted 2D contours with *H*_*c*_ and *H*_*u*_ scales for the DMP magnet (**a**) and SP magnet (**b**). (The converted software used to create the map is IGOR pro 6.10A, https://www.sciencesoftware.com.cn/Newsoftware_detail.aspx?sid=347&renqun_youhua=151434&bd_vid=8372655517236161471).

Figure 5a,b present the raw FORC measurements for both magnets. Figure 6a,b are the converted FORC 2-D contour diagrams corresponding to Fig. 5a,b. There is a small kink on the magnetization curve at the remanence for both of the magnets, indicating the presence of the surface-deteriorated magnetic layer due to the mechanical polishing^26^. While the surface-deteriorated layer has a minor effect on the magnetization reversal of the whole magnet. It is obvious that there is only one switching field peak at approximately 10 kOe for the SP magnet, while the FORC distribution for the DMP magnet possesses three switching field peaks with values around 6 kOe, 11 kOe and 12 kOe. The different peaks indicate the coexistence of three major reversal components in the DMP magnet, which correspond to the three kinds of matrix phases. The first one, located at 6 kOe, can be thought as the switching field of the (Pr/Nd_0.5_Ce_0.5_)_31_(Fe,TM)_68_B_1_ phase, while the second one and the third one at 11 kOe and 12 kOe are the switching fields of the (Pr/Nd_0.8_,Ce_0.2_)_31_(Fe,TM)_68_B_1_ and (Pr/Nd)_31_(Fe,TM)_68_B_1_ phases, respectively. In addition, we can see that the second switching field peak at about H_c_ = 11 kOe has a considerable vertical spread (spread in the bias distribution). It has been shown experimentally that spread in the bias distribution can be attributed primarily to dipole interactions^25,27^. This result reveals that the dipole interaction only appears between the adjacent (Pr/Nd_0.8_,Ce_0.2_)_31_(Fe,TM)_68_B_1_ phase, which is the shell of the other matrix phases. Moreover, the overlap of the second and third switching field peaks implies that the switching process of the (Pr/Nd_0.8_,Ce_0.2_)_31_(Fe,TM)_68_B_1_ shell involves a pinning interaction arising from the (Pr/Nd)_31_(Fe,TM)_68_B_1_ phase. This interior interaction is the other reason for the better coercivity of the DMP magnet.

## Conclusions

In this work, a superior magnetic performance of Ce substituting Pr/Nd in Nd–Fe–B permanent magnets was obtained use the DMP method. The enhanced coercivity for the DMP magnet is mainly due to the easier formation of the grain boundary phase encapsulating the matrix phase and reducing the exchange coupling between adjacent matrix grains. Furthermore, our results of FORC analyses suggests that the pinning interaction in the individual main phase grains, which possesses a core–shell structure in the DMP magnets, further improves the coercivity of the magnet. The development of multi-matrix phases is promising for manufacturing Ce-based permanent magnets in mass production, which may greatly promote the sustainability, balance and diversity of the global rare earth industry.

## Methods

### Magnet preparation

Following to the DMP method reported by our group, three type of alloys with the nominal composition of (Pr/Nd_1-x_Ce_x_)_31_(Fe,TM)_68_B_1_ (x = 0, 0.2 and 0.5, defined as alloy I, alloy II and alloy III, respectively) were prepared by induction melting and strip casting. The DMP magnet were fabricated by mixing the cast strips of alloy I and alloy III under the ratio of 3:2, which has the same nominal composition of alloy II (hereafter abbreviated as SP magnet). Then the mixed DMP and SP strips were subjected hydrogen decrepitation and jet-milling to obtain powders with average particle size of ~ 3.5 μm. The obtained powders were compacted after alignment under 2.0 T magnetic fields and cold isostatic pressed at 220 MPa. Finally, vacuum sintering was performed at temperature in the range of 980–1050 °C for 2 h.

### Measurements and characterizations

The magnetic properties of the samples were measured with an NIM-2000 hysteresis-loop instrument (by National Institute of Metrology, China). Backscattered electron (BSE) scanning electron microscopy (SEM) images were obtained using a field-emission scanning electron microscope (JSM-7001F) equipped with an energy-dispersive spectroscopy detector (EDS). For transmission electron microscope (TEM) analysis, the samples were cut into small discs with diameter of 3 mm, and then mechanically polished down to 30 µm. Then they were glued on a copper ring and further ion-milled by Gatan 691 precision ion polishing system. After the ion-milling process, the samples immediately transferred to a TEM holder to avoid air oxidation and contamination, and then inserted into a JEM-2000FX microscope for crystal structure identification. The First-Order-Reversal-Curves (FORCs) were collected using a VersaLab VSM (Quantum Design manufacture) with an auto-run script programmed by the Visual-Basic software.
